# Enhancement of Antioxidant Systems and Storability of Two Plum Cultivars by Preharvest Treatments with Salicylates

**DOI:** 10.3390/ijms18091911

**Published:** 2017-09-06

**Authors:** Alejandra Martínez-Esplá, María Serrano, Daniel Valero, Domingo Martínez-Romero, Salvador Castillo, Pedro J. Zapata

**Affiliations:** 1 Department of Agro-Food Technology, University Miguel Hernández, 03312 Orihuela, Spain; alexme_13@hotmail.com (A.M.-E.); daniel.valero@umh.es (D.V.); dmromero@umh.es (D.M.-R.); scastillo@umh.es (S.C.); 2 Department of Applied Biology, University Miguel Hernández, 03312 Orihuela, Spain; m.serrano@umh.es

**Keywords:** *Prunus salicina* Lindl, salicylic acid, acetylsalicylic acid, methyl salicylate, antioxidant enzymes, phenolics, antioxidant activity, quality, postharvest, ripening

## Abstract

In this research the effect of salicylic acid (SA), acetylsalicylic acid (ASA), and methylsalicylate (MeSA) treatments, applied as a foliar spray during on-tree plum development, on fruit quality attributes, bioactive compounds, antioxidant activity, and the activity of the antioxidant enzymes at harvest and after long-term cold storage was evaluated in two plum cultivars (“Black Splendor”, BS, and “Royal Rosa”, RR). At harvest, plum quality parameters, such as weight, total phenolics (including anthocyanins, in BS), total carotenoids, and antioxidant activity, in both hydrophilic and lipophilic compounds were found at higher levels in plums from SA-, ASA-, and MeSA-treated trees than in those from control trees. During storage, fruit firmness, total acidity, and antioxidant compounds were at higher levels in treated, than in control, plums, which show an effect of salicylate treatments on delaying the plum postharvest ripening process. In addition, the activity of the antioxidant enzymes catalase (CAT), peroxidase (POX), superoxide dismutase (SOD), and ascorbate peroxidase (APX) were also enhanced at the time of harvest in salicylate-treated plums as compared with plums from control trees. The activity of these antioxidant enzymes was also found at higher levels in salicylate-treated plums during storage. Thus, preharvest treatment with salicylates could be a safe, eco-friendly, and new tool to improve and maintain plum quality attributes, and especially their content of antioxidant compounds, with an additional effect on delaying the postharvest ripening process through increasing the levels of antioxidant compounds and the activity of the antioxidant enzymes.

## 1. Introduction

Oxidative stress in plant cells involves the accumulation of free radicals, especially reactive oxygen species (ROS), such as the superoxide radical (O_2_^−^), hydrogen peroxide (H_2_O_2_), and hydroxyl radical (OH^−^) [1]. ROS are inevitably generated in plant cells as a consequence of normal metabolism, mainly in reactions catalysed by oxidase and lipoxygenase, and in β-oxidation of fatty acids, and they are continuously eliminated by enzymatic and non-enzymatic systems. Thus, ROS content in plant cells is dependent on their producing systems and scavenging mechanism [1]. Non-enzymatic antioxidant compounds are reduced forms of ascorbate and glutathione, tocopherols, phenolics, alkaloids, and carotenoids, while enzymatic scavenging mechanisms mainly include superoxide dismutase (SOD), catalase (CAT), peroxidase (POD), and ascorbate peroxidase (APX) [1,2]. However, in spite of the presence of these efficient antioxidant systems, oxidative damage still occurs in plant cells either due to uncontrolled production or inefficient scavenging of ROS, which usually occurs under stress conditions and associated to senescence. In this sense, since the overall process of fruit ripening is considered as a functionally-modified protracted form of senescence, associated with ROS accumulation [2], the presence of a high content of antioxidant compounds and high activity of antioxidant enzymes could lead to a delay of the fruit postharvest ripening process and maintain fruit quality attributes for a long time.

Salicylic acid (SA) and its derivatives, acetyl salicylic acid (ASA) and methylsalicylic acid (MeSA), are nowadays considered as plant hormones with important roles in a wide range of physiological processes, such as inducing systemic acquired resistance (SAR), modulation of opening and closing of the stomatal aperture, flowering, seedling germination, and providing plant tolerance against different kinds of stress [3]. In addition, postharvest treatments with salicylates have been shown to reduce decay (by increasing fruit resistance to diseases) and chilling injury in numerous commodities, as well as to improve other quality properties, such as appearance, texture, and nutritional content [4,5]. Thus, postharvest treatments with SA maintained higher-quality attributes on apricots [6], as well as ASA and MeSA treatments on pomegranate [7,8], SA, and ASA treatments on sweet cherry [9], or ASA treatments on kiwifruit [10] among others. In addition, in mango and sugar apple, postharvest SA treatments led to lower superoxide free radical production and lipoxygenase activity and increases in the activity of SOD, CAT, and APX antioxidant enzymes during storage as compared with control fruits [11,12]. Similar increases in these antioxidant enzymes have been observed in peach fruits after postharvest SA treatment [13], which hasten cleaning of the superoxide radical generated during fruit ripening and, in turn, could be responsible for the effect of SA treatment on delaying the fruit ripening process. Specifically, in plums, it has been recently reported that SA postharvest treatments reduced chilling injury, softening, and colour evolution, leading to maintenance of fruit quality along storage at 20 °C or low temperature in “Santa Rosa” [14,15], “Satluj” [16], and “Qingnai” [17] cultivars.

However, as far as we know, there are no previous reports regarding the effect of preharvest treatments of plum trees with SA, ASA ,or MeSA on fruit quality attributes and antioxidant systems, at harvest or during storage, although some literature exists about this issue in other fruits. Thus, a combination of pre- and postharvest treatments of strawberry with SA decreased fungal decay and maintained overall quality during storage [18]. Similarly, preharvest foliar sprays of jujube plants with 2.0 mM SA decreased decay caused by *Alternaria alternata* and *Monillinia fructicola* either at harvest or during cold storage [19]. In sweet cherry SA treatment of trees three days before harvesting or at harvest date increased fruit resistance against *M. fructicola* and *Penicillium expansum* during storage, by increasing β-1,3-glucanase, phenylalanine ammonia-lyase (PAL) and POD activities [20]. Moreover, SA, ASA, or MeSA treatments of sweet cherry trees, at 0.5, 1.0, and 2.0 mM concentrations, respectively, applied at pit hardening, initial colour changes, and onset of ripening increased fruit quality attributes, such as weight, firmness, and content of total phenolics, total anthocyanins, and antioxidant activity [21,22,23].

The aim of this research was to evaluate the effect of SA, ASA, and MeSA preharvest treatments of plum trees on fruit quality attributes, antioxidant compounds, and the activity of the antioxidant enzymes, SOD, CAT, POD, and APX, at harvest and during cold storage for long periods.

## 2. Results

### 2.1. Fruit Quality Parameters

Fruit weight at harvest was 92.2 ± 2.3 and 68.6 ± 1.8 g in “Black Splendor” and “Royal Rosa” (BS and RR) control plums, respectively, and significantly higher in plums from salicylate-treated trees, these increases being ca. 10% in all treated plums, except in SA-treated BS plums, in which the highest effect was observed, with a 24% increase in fruit weight (Table 1). This increase on fruit weight led to a significant increase in total yield in both plum cultivars. Thus, the yield in BS was 33.18 ± 1.42 kg/tree in control trees and 39.25 ± 1.23, 35.81 ± 1.62, and 36.91 ± 1.71 kg/tree in trees treated with SA, ASA, and MeSA, respectively, and in RR values of 28.01 ± 1.39 kg/tree in control trees and 34.07 ± 1.71, 32.58 ± 2.09, and 31.42 ± 1.70 kg/tree in those treated with SA, ASA, and MeSA, respectively, were obtained. However, no significant effect was observed in the number of fruit per tree (data not shown). TSS at day 0 were 12.3–12.7 and 11.5–12 g/100 g in BS and RR plums, respectively, without significant differences among control and treated plums. Similarly, no effect of salicylate treatments was observed in TA at harvest, with values around 1.70 and 0.75 g/100 g in BS and RR, respectively, leading to fruits with similar RI values. Finally, colour hue was also similar in control and treated plums, with values ≈ 16.5 in BS and 26 in RR, as well as fruit firmness, which was 9.47 ± 0.37 and 5.86 ± 0.29 N·mm^−1^ in BS and RR cultivars, respectively (Table 1). During cold storage significant increases in RI were observed in all plum fruits, mainly due to decreases in TA, although these changes were significantly delayed in plums from treated trees. In a similar manner, colour evolution, manifested as decreases in colour hue, and softening were also significantly delayed in treated plums with respect to controls (Table 1). Weight loss increased during storage, reaching final values of ca. 5–7% in both plum cultivars without significant differences between control and treated plums (data not shown).

### 2.2. Bioactive Compounds and Antioxidant Activity

**Figure 1:**
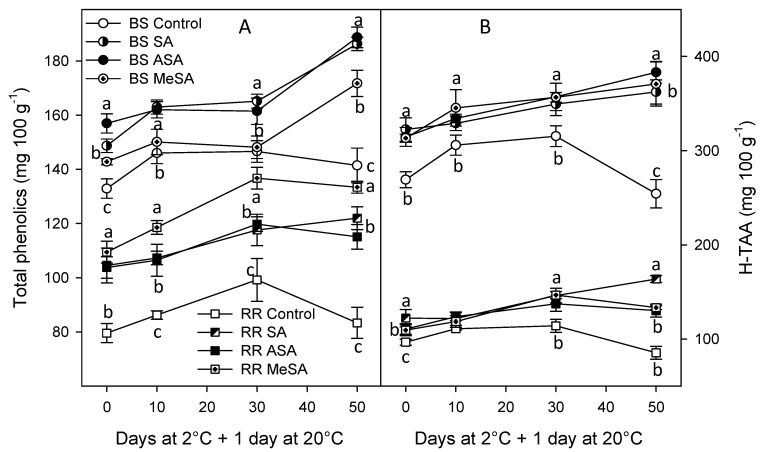
Total phenolic concentration (**A**) and hydrophilic antioxidant activity (H-TAA); (**B**) in “Black Splendor” (BS) and “Royal Rosa” (RR) plums from control and salicylic acid (SA)-, acetyl salicylic acid (ASA)-, and methyl salicylic acid (MeSA)-treated trees at harvest and during storage at 2 °C + 1 day at 20 °C. Symbols for BS and RR control and treated plums are similar for figures A and B. Data are the mean ± SE of three replicates. Different lowercase letters show significant differences (*p* < 0.05) among treatments for each sampling date and cultivar.

Total phenolic concentration in control BS and RR treated plums was 132.89 ± 3.59 and 79.58 ± 3.56 mg/100 g, respectively, at harvest and they increased until 30 days of storage, then a decrease occurred. However, in plums from salicylate-treated trees, phenolic concentrations were significantly higher and they increased until the last sampling date (50 days of cold storage + 1 day at 20 °C, the highest effect being found for SA and ASA treatment in BS plum and for MeSA treatment in RR plums (Figure 1). A similar trend was observed in the antioxidant activity of the hydrophilic extracts, H-TAA, which was significantly higher in all salicylate-treated plums than in controls, for most sampling dates (Figure 1).

**Figure 2:**
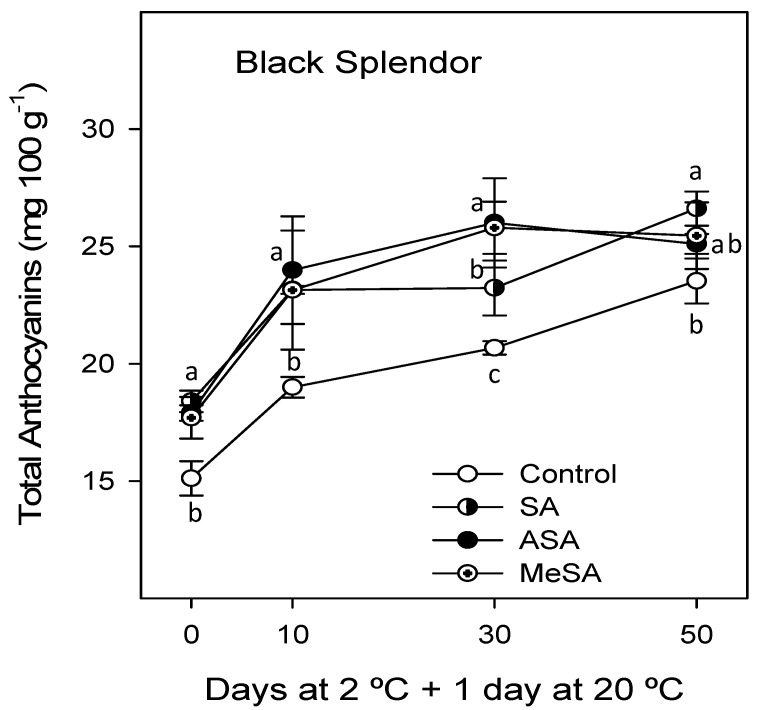
Total anthocyanin concentration in “Black Splendor” plums from control and salicylic acid (SA)-, acetyl salicylic acid (ASA)-, and methyl salicylic acid (MeSA)-treated trees at harvest and during storage at 2 °C + 1 day at 20 °C. Data are the mean ± SE of three replicates. Different lowercase letters show significant differences (*p* < 0.05) among treatments for each sampling date.

Total anthocyanin concentration was measured in the flesh of BS, which has a purple colour, due to anthocyanin pigments, while they are not found in the yellow cultivar, RR. However, carotenoids were measured in both cultivars, since they are the pigments responsible for the yellow colour of RR flesh, but are also present in the flesh of the purple cultivar, BS. Results show that all salicylate treatments significantly increased total anthocyanin concentration at harvest, although no significant differences were observed among SA-, ASA-, and MeSA-treated plums. During storage total anthocyanin concentration increased in control and treated plums, these concentrations being significantly higher in plums from salicylate-treated trees than in those from controls, except in the last sampling date (Figure 2). In the last sampling date anthocyanin concentration was still significantly higher only in fruit from SA-treated plums, with respect to those from the control.

**Figure 3:**
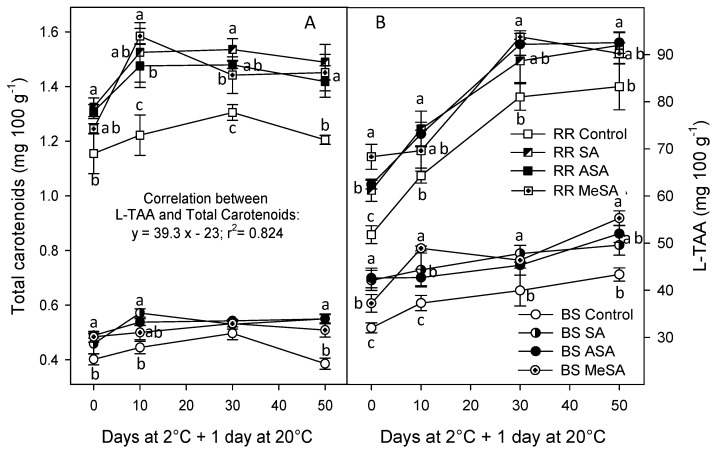
Total carotenoid concentration (**A**) and lipophilic antioxidant activity (L-TAA); (**B**), in “Black Splendor” (BS) and “Royal Rosa” (RR) plums from control and salicylic acid (SA)-, acetyl salicylic acid (ASA)-, and methyl salicylic acid (MeSA)-treated trees at harvest and along storage at 2 °C + 1 day at 20 °C. Symbols for BS and RR control and treated plums are similar for figures A and B. Data are the mean ± SE of three replicates. Different lowercase letters show significant differences (*p* < 0.05) among treatments for each sampling date and cultivar.

Total carotenoid concentration in control plums was 0.40 ± 0.02 mg/100 g in the BS cultivar, which has purple flesh and 1.15 ± 0.07 mg/100 g in RR, the cultivar with yellow flesh, at harvest. For both cultivars, carotenoid concentration enhanced, as did the storage time, until 30 days of storage and then a decrease trend occurred. A similar behaviour was found in plums from salicylate-treated trees, although in them carotenoid concentration was always higher than in controls (Figure 3A). The antioxidant activity in the lipophilic fraction of plum extracts also increased along storage, whose values were enhanced as a consequence of salicylate treatments, although without significant differences among plums from SA-, ASA-, or MeSA-treated trees (Figure 3B). In addition, taking into account data from both cultivars, treatments, and sampling dates, a high correlation was obtained between L-TAA and total carotenoids (*r*^2^ = 0.824). However, this correlation was lower if data from each cultivar were taken independently.

**Figure 4:**
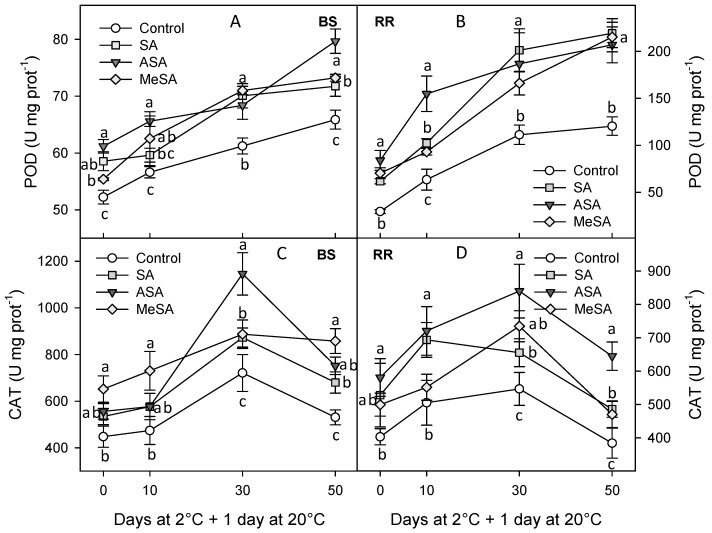
Activity of peroxidase (POD) for “Black Splendor” (BS) (**A**) and “Royal Rosa” (**B**) cultivars and catalase (CAT) for “Black Splendor” (BS) (**C**) and “Royal Rosa” (**D**) plum cultivars from control and salicylic acid (SA)-, acetyl salicylic acid (ASA)-, and methyl salicylic acid (MeSA)-treated trees at harvest and along storage at 2 °C + 1 day at 20 °C. Data are the mean ± SE of three replicates. Different lowercase letters show significant differences (*p* < 0.05) among treatments for each sampling date and cultivar.

**Figure 5:**
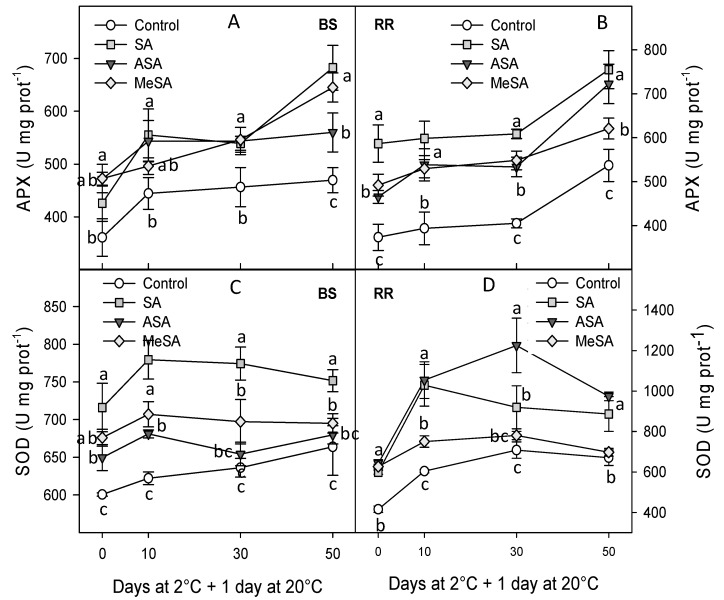
Activity of ascorbate peroxidase (APX) for “Black Splendor” (BS) (**A**) and “Royal Rosa” (**B**) cultivars and superoxide dismutase (SOD) for “Black Splendor” (BS) (**C**) and “Royal Rosa” (**D**) plum cultivars from control and salicylic acid (SA)-, acetyl salicylic acid (ASA)-, and methyl salicylic acid (MeSA)-treated trees at harvest and along storage at 2 °C + 1 day at 20 °C. Data are the mean ± SE of three replicates. Different lowercase letters show significant differences (*p* < 0.05) among treatments for each sampling date and cultivar.

The activity of the antioxidant enzymes POD, CAT, APX, and SOD at harvest and during storage was found at significantly higher levels in plums from salicylate-treated trees than in controls, at most sampling dates. However, in general, no significant differences among SA, ASA, and MeSA treatments could be observed. Thus, ASA was the most effective treatment in enhancing POD only at day 50 in BS and at day 10 in RR (Figure 4A,B) and, for CAT activity, the highest effect was found for ASA treatment at days 30 and 50 for BS and RR cultivars (Figure 4C,D). On the other hand, the highest increase in APX was obtained in SA- and MeSA-treated BS plums at day 50, and in RR from SA-treated trees at days 0, 10, and 30 (Figure 5A,B). Finally, for SOD, the most effective treatment was also SA in the BS cultivar during the whole storage period, but in the RR cultivar it was ASA treatment just in samples stored for 30 days (Figure 5C,D). However, it is worthy to note that even after 50 days of cold storage + 1 day at 20 °C the activity of these antioxidant enzymes was still higher in all treated plums than in controls.

## 3. Discussion

TSS, TA, colour, and firmness are the main important quality attributes in plums, although large variations in these parameters can be found depending on the cultivar, production area, climatic conditions, and harvest season [24,25]. However, plums are categorised as climacteric fruit with a limited postharvest storage life due to evolution of the ripening process leading to pigment changes, softening, increase in TSS and reduction in TA even if they are stored at cold temperature [24,25,26,27,28]. In the present study plums were harvested when reached their commercial ripening stage, according to size, external colour and TSS content characteristic for each cultivar. In turn, no significant differences on quality parameters were found as a consequence of salicylate treatments, except in fruit size, which was higher in plums from treated trees than in those from controls, as well as total tree yield. Accordingly, significant increases in cluster weight and yield were observed in vines sprayed with 1.5 or 2.0 mM SA, compared to control [29]. These results might indicate that treatments with salicylates could increase net photo-assimilate production of plum trees and/or the sink strength of developing fruits. In this sense, it has been reported that SA foliar application increased chlorophyll content, photosynthetic rate, and total dry weight in ginger plants [30], and increased activity of Rubisco and total yield have been also found after SA treatments in maize and mustard plants [31]. Along storage, fruit firmness, TA, and hue colour decreased while increases occurred in TSS and RI in plums from control and treated trees. However, these changes were significantly delayed (*p* < 0.05) in plums from salicylates treated trees with respect to controls (Table 1). Decreases in Hue colour index shows an intensification of purple colour of plum skin, which usually occurs during ripening, either on-tree or during storage [25,27]. On the other hand, increases in RI also show the evolution of the pre- and postharvest ripening process of plum fruits, as has been reported in a wide range of plum cultivars, either Japanese or European ones [27,28,32]. Taking into account that all trees (control and treated ones) were located in same farm, submitted to the same cultural practices (irrigation, fertilization, etc.) and under similar environmental conditions, these differences between plums from control and treated trees should be just attributed to the effect of salicylates treatments. Thus, a significant delay of the postharvest ripening process was obtained by salicylate treatments by delaying colour, firmness and RI changes. Similarly, it has been recently reported that postharvest SA treatment maintained quality parameters during storage in other plum cultivars, such as “Santa Rosa” [14,15] and “Satluj” [16]. Since plums are climacteric fruit [28], the effects of salicylate treatments on delaying postharvest ripening process and maintaining quality attributes could be mediated by a reduction of ethylene production. In fact, we have found that in BS and RR plums, preharvest treatments with SA, ASA, and MeSA decreased ethylene production with respect to control fruits, both at harvest and along storage time (unpublished data). Accordingly, reduced ethylene production has been observed after postharvest treatment with salicylates in a wide range of fruits, such as kiwifruit treated with ASA [33], and sugar apples [12], strawberries [18], and “Qingnai” plums treated with SA [17]. This effect of salicylates on inhibiting ethylene production has been attributed to the ability of salicylates of inhibiting 1-aminocyclopropane-1-carboxylic acid (ACC) synthase and ACC oxidase, the main enzymes involved in ethylene biosynthesis [33,34]. Moreover, Yin et al. [33] have recently reported that ASA may also interfere with ethylene perception.

Plums has been reported as a rich source of antioxidant compounds with health beneficial effects, such as phenolics (including anthocyanins), carotenoids and ascorbic acid, as compared with other fruits of the Mediterranean diet, although important differences in their concentration are found depending on cultivar [25,28,35,36,37,38]. It is interesting to note that SA, ASA, and MeSA preharvest treatment led to increased levels of total phenolic content, and total carotenoids in both plum cultivars at harvest, as well as total anthocyanin concentration in BS cultivar and these levels remained still at significantly higher concentration after 50 days of storage (Figure 2, Figure 3 and Figure 4). Moreover, H-TAA and L-TAA were also increased as a consequence of salicylate treatments and L-TAA was positively correlated with total carotenoid concentration. No previous reports are available regarding the effect of these treatments, either applied as post- or preharvest treatments, on carotenoid content or antioxidant activity on plums. Moreover, with respect to their effect on phenolic content just one paper has been previously published. In this report, SA postharvest treatment of “Santa Rosa” plum led to maintenance of higher total phenolic content during storage with respect to control fruits [14]. Nevertheless, some reports exist on other fruits for comparative purposes. Thus, preharvest treatments with SA, ASA, and MeSA of sweet cherry trees increased total phenolics, total anthocyanins and H-TAA of fruits at harvest and these differences were maintained along storage, leading to fruits with higher bioactive compounds and health beneficial effects with respect to controls [21,22,23,39]. Accordingly, SA preharvest treatments of table grape led to higher levels of these bioactive compounds at harvest and during postharvest storage [29]. On the other hand, postharvest treatments with SA, ASA, or MeSA maintained total phenolics, anthocyanins and antioxidant activity during cold storage in pomegranate [7,8], sweet cherry [9,40], cornelian cherry fruit [41], and apricot [6] and these enhancements were attributed to an increase of phenylalanine ammonia lyase activity, which is the main enzyme involved in the biosynthetic phenolic pathway. Since carotenoids, phenolics, including anthocyanins, and ascorbic acid have been proved to have beneficial effects against degenerative diseases [28,35,36,37] preharvest treatments with salicylates would provide healthier fruit for human consumption.

CAT, POD, APX, and SOD are the main antioxidant enzymes involved in the radical scavenging of ROS species and, thus, are considered as a mechanism for repairing cell oxidative damage [2,3]. The results of the present study show a general increase in these antioxidant enzymes during plum storage, although it is important to point out that these activities were higher in plums from salicylate-treated trees than in those from control trees. Accordingly, pre-harvest treatments with SA, ASA, or MeSA led to significant increases in these antioxidant enzyme activities in sweet cherry [23,39], and in peach, SOD, CAT, and POD activities were also higher during postharvest storage in SA-treated fruits [13]. In addition, postharvest treatments with SA were also effective on maintaining higher levels of SOD, CAT, and APX activities during storage of apricot, mango, and sugar apple fruit, which were accompanied by lower superoxide anion content and lower lipoxygenase activity [6,11,12]. Similar effects have been obtained in cucumber after SA postharvest treatment, alone or in combination with chitosan [42].

The increased activity of these antioxidant enzymes would lead to a more efficient cleaning of ROS species, such as O_2_^–^ OH, or ^1^O_2_, since they contribute to peroxidation of membrane lipids, damage to DNA and proteins, and acceleration of senescence processes [2,3,43,44]. Oxidative stress, involving ROS accumulation, has been associated with fruit ripening process, which is considered as a functionally-modified protracted form of senescence [2]. The ROS content in plant cell is dependent on their producing systems and scavenging mechanism, both enzymatic and non-enzymatic ones [1]. Non-enzymatic ROS scavenging are antioxidant compounds, such as reduced forms of ascorbate and glutathione, phenolics, alkaloids, and carotenoids, while enzymatic scavenging mechanisms include antioxidant enzymes, mainly SOD, CAT, POD, and APX. Thus, the effects of salicylate treatments on increasing the content of antioxidant compounds and the activity of antioxidant enzymes could lead to a delay of the fruit postharvest ripening process and to maintain plum quality attributes for longer periods, according to previous reports on guava and ber fruits [43,44]. However, this is the first study to show that SA, ASA, and MeSA treatments of plum trees increased the activities of CAT, POD, APX, and SOD at harvest and during postharvest storage, as well as the concentration of antioxidant compounds. Thus, the maintenance of plum quality properties, including bioactive compounds with antioxidant activity, along storage, in plums from treated fruits could be attributed to the enhanced activity of these antioxidant enzymes. Nevertheless, future sstudies are needed to corroborate the effect of salicylate treatments on reducing ROS and oxidative stress in plum fruits.

## 4. Materials and Methods

### 4.1. Plant Material and Experimental Design

In the present experiment two plum (*Prunus salicina* Lindl.) cultivars (“Black Splendor”, BS, and “Royal Rosa”, RR) were used, which were grown on a commercial farm (El Ciruelo) located at Cieza (Murcia, Spain) along the developmental cycle of 2014 spring–summer period. The date for full blossom was 22 February for both cultivars. The experiment was designed totally at random in triplicate by using three trees per replicate for each cultivar and treatment: control (distilled water), salicylic acid (SA) at 0.5 mM, acetylsalicylic acid (ASA) at 1 mM, and methyl salicylate (MeSA) at 0.5 mM, purchased from Sigma (Sigma-Aldrich, Madrid, Spain). These concentrations were chosen according to previous experiments in which 0.5, 1.0, and 2.0 mM doses of these compounds were applied, taking into account fruit yield and plum quality parameters at the time of harvest (unpublished data). Recently-prepared solutions (plus 0.5% of Tween-20 as surfactant) were foliar sprayed (3 L per tree) with a mechanical mist sprayer at three dates of the plum growth cycle: T1 (at pit hardening, 63 days after full blossom, DAFB), T2 (initial colour changes, 77 DAFB) and T3 (onset of ripening, 98 DAFB). These dates corresponded to key events in the plum fruit developmental process, according to previous experiments [25,45]. Plums were harvested at the commercial ripening stage based on fruit size, colour, and the content of total soluble solids (TSS) characteristic of each cultivar. The total yield was measured for each tree and expressed as kg per tree. Then, for each cultivar, treatment and replicate of about 100 plums were taken at random and used to calculate the fruit weight. These fruits were transferred to the laboratory and then four lots of 10 homogenous fruits were randomly performed and stored at 2 °C and relative humidity of 85% + 1 day at 20 °C to simulate retail conditions. Analytical determinations were made in recently-harvested fruits (day 0) and in stored fruits for 10, 30, and 50 days at 2 °C + 1 day at 20 °C by using one lot for each replicate and treatment taken at random from the store room.

### 4.2. Weight Loss, Firmness, Color, Total Soluble Solids (TSS), and Total Acidity (TA)

Each lot was weighed at day 0 and after the storage period and weight loss was expressed in percentage with respect to weight at harvest. Colour was measured at three equatorial points in each individual fruit by using a Minolta colorimeter CRC-200 (Oxaca, Japan). The 3 colour coordinates (L*, a* and b*) were recorded and colour expressed as Hue angle (arc tan b*/a*). For firmness, a TX-XT2i Texture Analyser (Stable Microsystems, Godalming, UK) was used and the force-deformation ratio necessary to achieve a 3% deformation of fruit diameter was measured, with the results expressed as N·mm^−1^. After that, the 10 fruits of each lot were peeled and the flesh cut in small pieces to obtain a homogeneous sample for each replicate. TSS were determined in duplicate from the juice obtained from 5 g of each sample with a digital refractometer (Atago PR-101, Atago Co. Ltd., Tokyo, Japan) at 20 °C, and expressed as g/100 g. TA was determined in duplicate with the same juice by automatic titration (785 DMP Titrino, Metrohm) with 0.1 N NaOH up to pH 8.1, using 1 mL of diluted juice in 25 mL distilled H_2_O, and the results expressed as g malic acid equivalent 100 g^−1^ fresh weight. The ripening index (RI) was calculated as the ratio TSS/TA.

### 4.3. Bioactive Compounds and Antioxidant Activity

Total phenolics were extracted according to Tomás-Barberán and Espín [46], by homogenizing 5 g of fruit flesh tissue (after peeling the fruit) and 10 mL of water:methanol (2:8) containing 2 mM NaF (to inactivate polyphenol oxidase activity and prevent phenolic degradation). Then the extract was centrifuged at 15,000× *g* for 15 min at 4 °C. Total phenolics were quantified in duplicate in the supernatant by using Folin-Ciocalteu reagent, according to Singleton et al. [47] and results were expressed as mg gallic acid equivalent 100 g^−1^ on a fresh weight basis. Total anthocyanin concentration was determined according to the method of García-Viguera et al. [48], adapted as previously reported [49]. Total anthocyanin concentration was calculated using cyanidin-3-glucoside (molar absorption coefficient 23,900 L·cm^−1^·mol^−1^, molecular weight 449.2 g·mol^−1^) and results were expressed as mg/100 g fresh weight. Total antioxidant activity (TAA) was quantified according to the method previously described by Arnao et al. [50] and modified by Díaz-Mula et al. [27], which enables determination of TAA due to both hydrophilic (H-TAA) and lipophilic (L-TAA) compounds in the same extraction. Briefly, 5 g of flesh tissues were homogenized in 5 mL of 50 mM phosphate buffer pH 7.8 and 3 mL of ethyl acetate, and then centrifuged at 15,000× *g* for 15 min at 4 °C. The upper and lower fractions were used for L-TAA and H-TAA quantification (both made in duplicate), respectively, by using the enzymatic system composed of the chromophore 2,2-azino-bis-(3-ethylbenzothiazoline-6-sulfonic acid) di-ammonium salt (ABTS), horseradish peroxidase enzyme (HRP) and its oxidant substrate (hydrogen peroxide). A calibration curve was performed with Trolox ((*R*)-(+)-6-hydroxy 2,5,7,8,-tetramethyl- croman-2-carboxylic acid) (0 to 20 nmol) from Sigma (Madrid, Spain) and results were expressed as mg/100 g Trolox equivalents on a fresh weight basis. Total carotenoids were estimated in the lipophilic extract, as previously reported by Arnao et al., [50] by reading the absorbance at 450 nm in a UNICAM Helios-spectrophotometer (Cambridge, UK), and expressed as mg/100 g.

### 4.4. Antioxidant Enzymes

Crude extracts for POD, CAT, APX, and SOD enzymes were performed by homogenizing 5 g of plum samples (fresh tissue, after peeling the fruit) with 10 mL of phosphate buffer 50 mM, pH 7.0, containing 1% (*w*/*v*) polyvinyl-pyrrolidone and 1 mM ethylen-diamine-tetraacetic acid (EDTA). The homogenate was centrifuged at 15,000× *g* for 30 min at 4 °C and the supernatant was used for antioxidant enzyme assays as previously reported [51,52]. POD was measured according to Luo et al. [17] in a reaction mixture containing 50 mM phosphate buffer pH 7.0, 12 mM H_2_O_2_, 14 mM guaiacol, and 100 µL of enzymatic extract in a total volume of 3 mL. The increase of absorbance at 470 nm, due to guaiacol oxidation, was measured for 1 min. One enzymatic unit (U) was defined as a 0.01 absorbance increase per min, and peroxidase activity was expressed as U·min^−1^·mg protein^−1^. For CAT and APX activities, the protocol described by Zhang et al. [10] was used. In brief, for CAT activity the reaction mixture contained 100 µL of the above extract and 2.9 mL 50 mM phosphate buffer pH 7.0, containing 15 mM H_2_O_2_ and the degradation of H_2_O_2_ was measured by the decrease of absorbance at 240 nm during 1 min. One enzymatic unit (U) was defined as a 0.01 absorbance decrease per minute, and CAT activity was expressed as U·min^−1^·mg protein^−1^. For APX quantification the assay mixture contained 50 mM potassium phosphate pH 7.0, 0.5 mM ascorbic acid, 1 mM H_2_O_2_ and 100 µL of crude extract. The decrease of absorbance at 290 nm during 1 min was measured and one enzymatic unit of APX (U) was defined as the amount of enzyme that oxidizes 1 mmol of ascorbate per minute, expressed as U·min^−1^·mg protein^−1^. SOD activity was determined photochemically, as described in Zhang et al. [10], with slight modifications. The reaction solution contained 50 mM phosphate buffer, pH 7.8, 5 mM methionine, 100 mM EDTA and 65 mM nitro-blue-tetrazolium (NBT). To 2.9 mL of this solution 25 µL of enzyme extract and 40 µL of 0.15 mM riboflavin were added. The tubes were then placed in a fluorescent light incubator (40 W, 10 min) and the formation of blue formazan was monitored by recording the absorbance at 560 nm. One unit (U) of SOD activity is defined as the amount of enzyme that causes a 50% inhibition of NBT reduction under the assay conditions. The results are reported as U mg protein^−1^ min^−1^. Total protein content in the crude extract was quantified according to Bradford [53].

### 4.5. Statistical Analysis

Experimental data were subjected to ANOVA analysis. Sources of variation were treatments and storage. The overall least significant differences (Fisher”s LSD procedure, *p* < 0.05) were calculated and used to detect significant differences among treatments and storage time. All analyses were performed with SPSS software package v. 11.0 for Windows (SPSS, 2001, IBM Corporation, Armonk, NY, USA). Linear regressions were performed between total antioxidant activity (either hydrophilic or lipophilic) and phenolic and carotenoid contents, taking into account all sampling data.

## 5. Conclusions

Preharvest SA, ASA, and MeSA treatments of plum trees would have commercial interest with low costs (an average of USD $30 per hectare) resulting in great benefits to fruit quality and plum yield. In addition, softening, colour evolution, and acidity losses during storage occurred at lower rates in treated, than in control, plums, which were attributed to the effect of salicylate treatments on delaying the postharvest ripening process. Concentrations of antioxidant compounds (total phenolics and total carotenoids) were also enhanced by salicylate treatments, which are responsible for the increase in H-TAA and L-TAA. Moreover, these treatments increased the activity of the antioxidant enzymes CAT, POD, APX, and SOD, which, together with the enhancement of the antioxidant compounds, could contribute to eliminate ROS efficiently and, in turn, to delay postharvest ripening and senescence processes, extending the shelf life of plum fruits. Thus, preharvest treatments with salicylates could be a safe, eco-friendly, and new tool to improve and maintain plum quality attributes, and especially the content on antioxidant compounds with beneficial health effects.

## Figures and Tables

**Table 1:** Fruit weight, yield, and quality parameters at harvest and changes during storage (from day 0 to day 50) for total soluble solids (TSS), total acidity (TA), ripening index (RI) and colour (hue angle) in “Black Splendor” (BS) and “Royal Rosa” (RR) plums from control and salicylic acid (SA, 0.5 mM)-, acetyl salicylic acid (ASA, 1 mM)-, and methyl salicylate (MeSA, 0.5 mM)-treated trees *.

Parameter	Days	Control	SA	ASA	MeSA
Weight (g)					
BS	Day 0	92.2 ± 2.3 c	114.9 ± 4.3 a	102.1 ± 3.4 b	99.6 ± 2.6 b
RR	Day 0	68.6 ± 1.8 b	76.5 ± 1.9 a	77.5 ± 1.6 a	76.5 ± 1.8 a
Yield (kg/tree)					
BS	Day 0	33.18 ± 1.42 c	39.25 ± 1.23 a	35.81 ± 1.62 b	36.91 ± 1.71 b
RR	Day 0	28.01 ± 1.39 b	34.07 ± 1.71 a	32.58 ± 2.09 a	31.42 ± 1.70 a
TSS (g/100 g)					
BS	Day 0	12.73 ± 0.21 A,a	12.40 ± 0.15 A,a	12.49 ± 0.21 A,a	12.30 ± 0.11 A,a
	Day 50	13.32 ± 0.12 B,a	12.70 ± 0.13 A,b	12.75 ± 0.10 A,b	12.73 ± 0.19 A,b
RR	Day 0	11.97 ± 0.11 A,a	11.50 ± 0.11 A,a	11.65 ± 0.24 A,a	11.78 ± 0.15 A,a
	Day 50	12.85 ± 0.31 B,a	11.85 ± 0.20 A,b	11.93 ± 0.22 A,b	12.03 ± 0.11 A,b
TA (g/100 g)					
BS	Day 0	1.67 ± 0.04 A,a	1.77 ± 0.03 A,a	1.64 ± 0.04 A,a	1.76 ± 0.08 A,a
	Day 50	0.93 ± 0.02 B,c	1.34 ± 0.02 B,b	1.30 ± 0.03 B,b	1.56 ± 0.02 B,a
RR	Day 0	0.75 ± 0.03 A,a	0.79 ± 0.02 A,a	0.74 ± 0.02 A,a	0.76 ± 0.03 A,a
	Day 50	0.41 ± 0.02 B,b	0.64 ± 0.02 B,a	0.59 ± 0.02 B,a	0.63 ± 0.02 B,a
RI (TSS/TA)					
BS	Day 0	7.26 ± 0.15 A,a	7.56 ± 0.11 A,a	7.05 ± 0.31 A,a	6.99 ± 0.24 A,a
	Day 50	14.32 ± 0.55 B,a	9.76 ± 0.14 B,b	9.51 ± 0.22 B,b	8.16 ± 0.22 B,c
RR	Day 0	15.96 ± 0.41 A,a	15.75 ± 0.36 A,a	15.74 ± 0.57 A,a	15.50 ± 0.33 A,b
	Day 50	31.34 ± 0.55 B,a	18.52 ± 0.35 B,c	20.22 ± 0.33 B,b	19.09 ± 0.55 B,b
Colour (Hue)					
BS	Day 0	16.52 ± 0.56 A,a	17.09 ± 0.78 A,a	16.40 ± 0.26 A,a	16.81 ± 0.46 A,a
	Day 50	10.49 ± 0.64 B,c	12.42 ± 0.91 B,b	12.51 ± 0.33 B,b	14.28 ± 0.23 B,a
RR	Day 0	25.26 ± 0.21 A,a	25.21 ± 0.47 A,a	26.56 ± 0.22 A,a	26.79 ± 0.22 A,a
	Day 50	18.55 ± 0.14 B,c	23.14 ± 0.67 B,b	23.98 ± 0.24 B,b	25.97 ± 0.21 B,a
Firmness (N·mm^−1^)					
BS	Day 0	9.47 ± 0.37 A,a	9.96 ± 0.41 A,a	9.87 ± 0.0.30 A,a	9.56 ± 0.39 A,a
	Day 50	3.34 ± 0.14 B,b	4.62 ± 0.0.16 B,a	4.69 ± 0.36 B,a	4.37 ± 0.17 B,a
RR	Day 0	5.86 ± 0.29 A,a	7.72 ± 0.23 A,a	5.92 ± 0.26 A,a	6.05 ± 0.32 A,a
	Day 50	2.82 ± 0.16 B,b	3.92 ± 0.23 B,a	4.06 ± 0.20 B,a	3.95 ± 0.15 B,a

* For each parameter and plum cultivar different capital letters denote significant differences (*p* < 0.05) over storage, while lowercase letters denote significant differences (*p* < 0.05) among treatments for each sampling date and cultivar.
